# Monolithic carbon xerogel with co-continuous hierarchical porosity *via* one-step, template- and catalyst-free hydrothermal reaction with resorcinol and formaldehyde†

**DOI:** 10.1039/c9ra00904c

**Published:** 2019-03-26

**Authors:** Hyoung-Ju Yoon, Jae Young Lee, Jae-Suk Lee, Tae-Ho Yoon

**Affiliations:** School of Materials Science and Engineering, Gwangju Institute of Science and Technology (GIST) 123 Cheomdan-gwagiro (Oryong-dong), Buk-gu Gwangju 61005 Republic of Korea thyoon@gist.ac.kr

## Abstract

Monolithic carbon xerogel (MCX) with co-continuous hierarchical porosity was prepared *via* a one-step, template- and catalyst-free hydrothermal polycondensation reaction with resorcinol (R), formaldehyde (F) and distilled water (W), followed by pyrolysis and CO_2_ activation. The reaction was carried out in a pressurized Teflon mold at 100 °C for 6 h, while F/R (2.2, 2.4, 2.6, and 2.8) and R/W ratios (40 and 45) were varied to obtain a co-continuous pore structure with interconnected particles. Next, the gels were dried at 60 °C for 36 h and then at 100 °C for 12 h to produce xerogels, which were then subjected to pyrolysis at 900 °C for 2 h and CO_2_ activation at 1000 °C for 2, 4 or 6 h. A co-continuous pore structure with interconnected particles was observed in gels with F/R = 2.4 and 2.6 at R/W = 40 and with F/R = 2.2 at R/W = 45, but the gel with F/R = 2.4 at R/W = 40 was the only one that showed no crack generation upon 6 h CO_2_ activation. Thus, this gel was subjected to a N_2_ sorption study, which resulted in a specific surface area (SSA) of 1418, 2489 and 3418 m^2^ g^−1^ at 2, 4 and 6 h activation, respectively. This was attributed to the introduction of micro-pores *via* activation, which also generated meso- and macro-pores to form hierarchical porosity.

## Abstract 2

Monolithic carbon xerogel (MCX) with co-continuous hierarchical porosity was prepared *via* a one-step, template- and catalyst-free hydrothermal polycondensation reaction with resorcinol, formaldehyde and distilled water.

## Graphical abstract

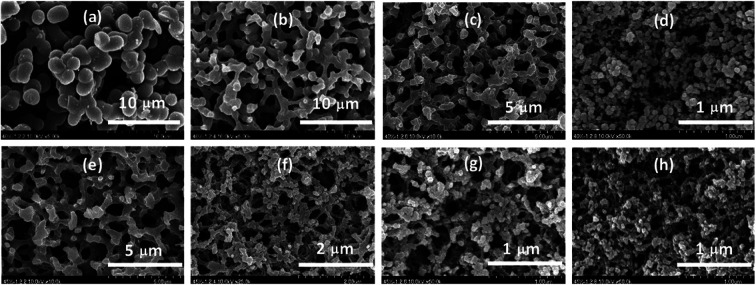

## Introduction

Porous carbon materials (PCMs) have received great attention in the filtration of water or air for an extended period, and their application has been recently extended to other areas such as electrodes for capacitors and batteries,^1,2^ catalyst support,^3^ and carbon dioxide capture.^4^ For such applications, a key requirement is monolithic structure^5,6^ with co-continuous^7,8^ and hierarchical porosity^9,10^ for easier handling and higher performance. In addition, interconnected carbon particles, rather than aggregated ones, for the pore structure are also desirable. Of course, high specific surface area (SSA) is also essential for better performance,^11,12^ while a simple process is desirable for low cost, such as a one-step^13^ template-free reaction^14^ without supercritical conditions or freeze drying.^15^

Unfortunately, the monolithic PCM was not available until Pekala introduced the base-catalyzed polycondensation reaction of resorcinol (R) and formaldehyde (F).^16^ It was one-step, template-free reaction in a glass ampule at 85 °C for 7 days. But solvent exchange and supercritical drying were employed to retain the pore structure after drying, and the monolithic carbon aerogel (MCA) was produced instead of monolithic carbon xerogel (MCX). Furthermore, the pores were formed by aggregated carbon particles, instead of interconnected particles, which is related to the rate of gelation and phase separation for spinodal decomposition.^17^ Pekala's study also showed low specific surface area (SSA) (500–700 m^2^ g^−1^) despite carrying out supercritical drying.

In 2008, however, Baumann and co-workers^18^ prepared MCAs with a high SSA of 3125 m^2^ g^−1^ by using a similar approach to Pekala's, but CO_2_ activation was employed, which is known to introduce micro-pores that lead to high SSA. In fact, there are a number of other studies reporting monolithic MCAs with high SSA values *via* CO_2_ activation.^19–22^ In Baumann's study, the obtained pore structure with aggregated carbon particles was converted to a co-continuous pore structure with interconnected particles *via* CO_2_ activation. Additionally, the CO_2_ activation also introduced meso- and macro-pores, leading to the formation of hierarchical porosity that is highly desirable for high performance. However, one critical drawback of this study is the aforementioned supercritical drying.

The MCX was prepared without supercritical drying by Tsuchiya^19^ through a similar approach used by Baumann^18^ but it required a multi-step process. As expected from 6 h of CO_2_ activation, a high SSA of 2965 m^2^ g^−1^ was reported, but the pore structure was formed by aggregated carbon particles and was similar to the one observed by Baumann.^18^ On the other hand, the MCX having a co-continuous pore structure with interconnected carbon particles was reported by Huang and co-workers.^23^ As noted, such unique pore structure can be attributed to (1) hydrothermal reaction at 100 °C, which is higher than 85 °C used by Pekala,^16^ or 80 °C used by Baumann,^18^ thus, leading to a higher degree of condensation reaction, and (2) phenol resin, which is much less soluble in water than in formaldehyde, thus, changing the degree of spinodal decomposition. In addition, the phenol resin may form much more rigid chemical bonds with formaldehyde than with resorcinol, producing a tenacious pore structure to afford xerogels without employing supercritical drying. Unfortunately, a low SSA of ∼620 m^2^ g^−1^ was reported despite the use of soft templates in the absence of CO_2_ activation. Similar pore structures were also reported by Liang and Dai,^24^ and Werner,^25^ but they also have some drawbacks to be resolved.

Among the aforementioned studies, the one by Bauman^18^ appears to be the most attractive since it involves a simple one-step, template-free reaction. Also, the resorcinol used has a much better solubility in water than in phenol, making an aqueous solution process possible, and is much less expensive than phloroglucinol, making it a very attractive resin for MCAs.^26^ Moreover, CO_2_ activation was successfully employed, which introduced not only micro-pores for a greatly increased SSA, but also meso- and macro-pores for hierarchical porosity. However, there are two major drawbacks to be resolved: aggregated carbon particles obtained rather than interconnected ones, and the aerogel produced rather than the xerogel.

Thus, in our previous study,^27^ Baumann's method was modified by employing a hydrothermal reaction at 100 °C, and by varying the ratios of resorcinol to water (R/W) and resorcinol to catalyst (R/C). As expected, this approach resulted in MCXs with a co-continuous pore structure *via* interconnected carbon particles, and with high enough rigidity to make supercritical drying unnecessary. In addition, subsequent CO_2_ activation provided a SSA of 1418, 2489 and 3311 m^2^ g^−1^ with 2, 4 and 6 h, respectively, along with hierarchical porosity.^27^ Nevertheless, there remained one critical concern; poor reproducibility due to the strong dependence of the pore structure on the catalyst concentration, which was also reported by others.^28–32^ This led to studies on a catalyst-free reaction,^33,34^ but a co-continuous pore structure has yet to be obtained.

In this study, therefore, a catalyst-free reaction was attempted with resorcinol and formaldehyde, which were combined in one-step, template-free hydrothermal reaction at 100 °C. The ratios of F/R (2.2, 2.4, 2.6 and 2.8) and R/W (40 and 45) were varied to obtain a co-continuous pore structure having interconnected carbon particles without employing supercritical drying. The xerogels were then subjected to pyrolysis and CO_2_ activation to provide high SSA with hierarchical porosity, and the gels were characterized by FE-SEM and N_2_ adsorption–desorption measurements before and after activation.

## Experimental

### Materials and methods

Resorcinol (R, Aldrich 398047, >99%) and formaldehyde (F, Aldrich 252549, 37 wt% in H_2_O) were used as-received to prepare monolithic carbon xerogels (MCXs) with co-continuous hierarchical pore structure *via* one-step polycondensation hydrothermal reaction,^16,21,36^ without using either catalysts or templates. Briefly, formaldehyde (F) was charged into a 50 ml glass vial by weight, followed by the addition of distilled water (W, resistivity of 18 MΩ cm *via* Milli-Q Advantage System) and then resorcinol (R). The total amount of water was controlled to 10 ml, which included the volume from the 37% formaldehyde (methanol was considered water). Based on 10 ml of distilled water, 4.5 or 4 g of R was added to obtain R/W = 40 or 45, respectively, and the monolithic RF xerogels were produced using the model by Scherdel and co-workers.^31^ In addition, the F/R ratio was also varied from 2.2 to 2.4. 2.6 or 2.8 to obtain a co-continuous pore structure *via* spinodal decomposition. Upon complete dissolution, the mixture was poured into a Teflon mold with four holes (36 mm in diameter and 40 mm in depth) and an aluminum top plate to prevent the pressure loss. Next, it was reacted in an air convection oven at 100 °C for 6 h, as reported previously.^27^ Finally, the resulting RF gels were dried in the mold for 36 h at 60 °C and for additional 12 h at 100 °C to achieve complete drying without crack generation.

A tube furnace (MSTF-1100, Myungsung Eng., Korea) was used for the pyrolysis of RF xerogels (diameter of ∼34 mm and thickness of ∼15 mm). It was heated from room temperature (RT) to 900 °C at 5 °C min^−1^ under a N_2_ flow of 200 sccm, and then held for 2 h at 900 °C, followed by natural cooling to RT under N_2_ flow. The carbon gels were cut in half and subjected to activation in the tube furnace at 1000 °C under a CO_2_ flow of 200 sccm for 2, 4 or 6 h. First, dimensional changes in the gels were estimated by size measurements, while crack generation was monitored by visual inspection since it is an important factor for the monolithic PCM. Next, the burn off ratio (BOR) was calculated based on the weight before and after activation.^19^ The MCXs were then analyzed by Field-Emission Scanning Electron Microscopy (FE-SEM, Jeol, JSM-7500F) at 10 keV with carbon coating, and their pore characteristics were studied with an adsorption instrument (ASAP-2010, Micromeritics, USA) using N_2_ at 77 K. The specific surface area (SSA) and pore size distribution were calculated by Brunauer–Emmett–Teller (BET) and Barrett–Joyner–Halenda (BJH) methods, respectively. The total pore volume was calculated from the total single point adsorption of pores with a radius less than 300 nm at *P*/*P*_o_ = 0.99, and the micro-pore volume was obtained by the *t*-plot theory.

## Results and discussion

### Preparation of monolithic RF xerogels

Monolithic RF gels were successfully prepared *via* one-step, template-free and catalyst-free polycondensation hydrothermal reaction with resorcinol and formaldehyde for all F/R (2.2, 2.4, 2.6 and 2.8) and R/W ratios (40 and 45). Subsequent drying at 60 °C for 36 h and for additional 12 h at 100 °C resulted in disk-shaped RF xerogels without cracks, as shown in Fig. S1,† which were similar to what was reported earlier.^27^ The successful preparation of xerogels can be attributed to the hydrothermal reaction using a Teflon mold at 100 °C,^23^ which produced hard RF gels that prevented the collapse of pores upon drying at 60 and 100 °C. A small shrinkage upon drying (a decrease in diameter from 36 to 34 mm) made it easier to remove the gels from the mold. The RF xerogels were rigid enough to resist manual breakage.

### Pyrolysis of monolithic RF xerogels

**Fig. 1:**
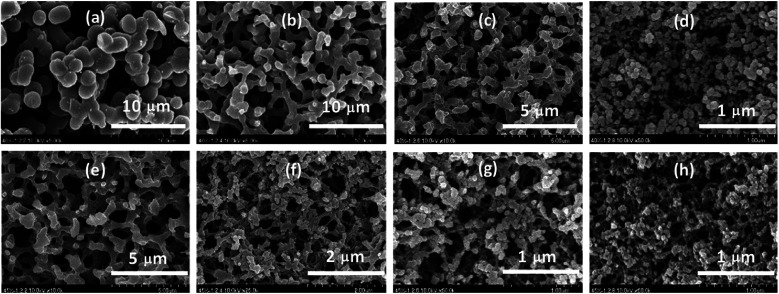
FE-SEM micrographs of RF xerogels after pyrolysis from R/W = 40 with F/R ratios of (a) 2.2, (b) 2.4, (c) 2.6, and (d) 2.8, and from R/W = 45 with F/R ratios of (e) 2.2, (f) 2.4, (g) 2.6 and (h) 2.8.

The monolithic carbon xerogels (MCXs) obtained by the pyrolysis of RF xerogels showed no shape change, but ∼50% weight loss and ∼50% volume shrinkage, regardless of the F/R and R/W ratios, which is similar to what was reported earlier.^22,32^ Despite such weight loss or shrinkage, the MCX was still hard enough to withstand manual breakage. FE-SEM micrographs from the MCXs at R/W = 40 revealed two different pore structures; a co-continuous pore structure with interconnected particles (F/R = 2.4 and 2.6) or aggregated particles (F/R = 2.2 and 2.8), as shown in Fig. 1. It can be seen that the carbon gel from F/R = 2.2 exhibited a large particle size of ∼3 μm, which decreased to ∼1, ∼0.5 and ∼0.1 μm for F/R = 2.4, 2.6 and 2.8, respectively. This decrease can be attributed to faster reactions with higher F/R ratios, and thus, higher viscosity of the reaction mixture, which in turn impedes the spinodal decomposition. The pore size also decreased with the increasing F/R ratio for the same reason, resulting in a smaller particle size. Such a trend is similar to what was reported with smaller R/C ratios.^30,32^ The MCX from R/W = 45 also exhibited two types of pore structure (Fig. 1), similar to R/W = 40. However, the co-continuous pore structure with interconnected particles was observed from F/R = 2.2, and the pore structure with aggregated particles from F/R = 2.4, 2.6 and 2.8. The difference between R/W = 45 and 40 can be attributed to the higher viscosity of the former (4.5 g R), resulting in slower spinodal decomposition, which in turn led to smaller particles and more aggregation.^17^

**Fig. 2:**
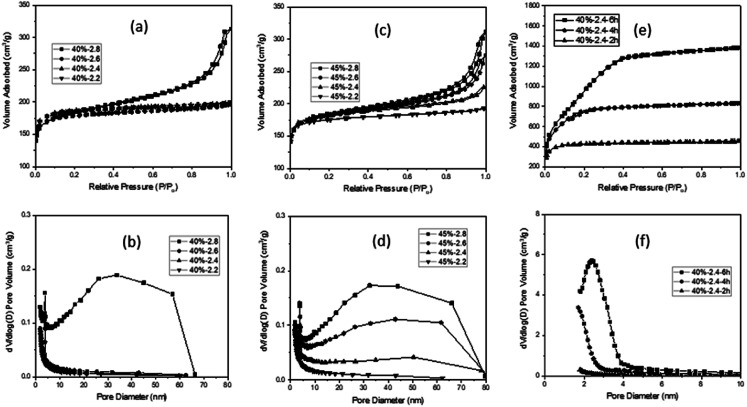
N_2_ isotherms of carbon xerogels from R/W = 45 (a) and R/W = 40 (c) and activated carbon xerogels from R/W = 40 (e) along with their pore size distribution (b, d and f).

In the N_2_ sorption analysis, the MCX from R/W = 40 exhibited a Type I isotherm (F/R = 2.2, 2.4, and 2.6), with the exception of F/R = 2.8 which showed a mixed isotherm of Type II and Type IV (Fig. 2a). The latter can be explained by the existence of micro-, meso- and macro-pores, which formed hierarchical porosity.^9^ This was also supported by the pore size distribution, as shown in Fig. 2b. The micro-pores are believed to be formed by pyrolysis, while the meso- and macro-pores are created by the aggregation of particles (Fig. 1d). The MCXs from F/R = 2.4 and 2.6 showed a Type I isotherm and the presence of only micro-pores from pyrolysis, since there was no aggregation of particles to form meso- and macro-pores (Fig. 1b and c). However, the MCX from F/R = 2.2 at R/W = 40 showed a Type I isotherm despite the presence of aggregated carbon particles (Fig. 1a). This is contrary to what was expected, since aggregated particles would show meso and macro-pores between the particles, as observed from the gel with F/R = 2.8. The discrepancy can be explained by the large particles which form only large pores upon aggregation, which are too large to be detected by the adsorption instrument (ASAP-2010, Micromeritics) that has a detection limit of 300 nm.

The MCXs from R/W = 45 also exhibited a similar trend, with one showing a Type I isotherm (F/R = 2.2) and three showing a mixed isotherm of Type II and Type IV (F/R = 2.4, 2.6 and 2.8) (Fig. 2c), as expected from the FE-SEM analysis. In addition, the N_2_ adsorption in the meso- and macro-pore range increased gradually with higher F/R ratios (2.4, 2.6 and 2.8). This is because the number of meso- and macro-pores increased with higher F/R ratios, which can be attributed to the increased number of small particles as the F/R ratio increased. This was supported by the pore size distribution (Fig. 2d), while the smaller size of particles with higher F/R ratios, which leads to higher aggregation, is evidenced by FE-SEM micrographs (Fig. 1).

It is interesting to note that the SSA decreased slightly with higher F/R ratios; 645, 641, 623 and 618 m^2^ g^−1^ with F/R = 2.2, 2.4, 2.6 and 2.8 at R/W = 40, respectively (Table 1). A similar trend was also observed with R/W = 45, as shown in Table S1.† This decrease may be related to the smaller particle size and higher degree of aggregation with the higher F/R ratio (FE-SEM in Fig. 1). As noted, these SSAs are similar to those reported earlier^20,24,35^ and appear to be the maximum values when activation or template is not utilized. On the other hand, the peak observed at 3–4 nm in all MCXs was attributed to the tensile strength effect caused by the ink-bottle or cylinder-type pores.^36^

**Table 1:** Characteristics of monolithic carbon xerogels without and with activation

	*S* a (m^2^ g^−1^)	*V*_total_ b (cm^3^ g^−1^)	*V*_micro_ c (cm^3^ g^−1^)	dBOR (%)
40-2.2	645 ± 12	0.252	0.242 (96.0%)	
40-2.4	641 ± 14	0.254	0.243 (95.7%)	
40-2.6	623 ± 12	0.271	0.232 (85.6%)	
40-2.8	618 ± 11	0.401	0.206 (51.4%)	
40-2.4-2 h	1481 ± 22	0.58	0.564 (97.2%)	35
40-2.4-4 h	2489 ± 25	1.067	1.025 (96.1%)	60
40-2.4-6 h	3418 ± 48	1.774	1.554 (87.6%)	79

a Specific surface area.

b Total pore volume.

c Micro pore volume.

d Burn off ratio from weight loss.

### CO_2_ activation of carbon xerogels

Activation of the MCX from R/W = 40 under CO_2_ flow resulted in a decreased sample size, and the decrease was larger for longer activation time and higher F/R ratio.^27^ But it was not quantitatively evaluated due to the difficulty of measurement. The activation also resulted in weight loss, which was expressed as the burn off ratio (BOR). The BOR increased again with activation time (Table S2†), as reported previously.^18,37^ For example, the MCX from F/R = 2.2 exhibited a BOR of 37, 63 and 80% with 2, 4 and 6 h, respectively. These values can be compared with 29, 48 and 70% obtained from F/R = 2.8 with 2, 4 and 6 h, respectively. A similar trend was reported by Tsuchiya.^19^ As noted, the weight loss decreased with higher F/R ratios (Table S2†), which can be attributed to the smaller pore size obtained with higher F/R ratios, as seen from the FE-SEM (Fig. 1), making it difficult for CO_2_ to penetrate into the porous structure. The MCX from R/W = 45 exhibited a similar trend (Table S3†), but the weight loss is slightly smaller than that from R/W = 40 at a given F/R ratio. This can once again be attributed to the smaller pore size from R/W = 45, and thus, decreased CO_2_ penetration into the pores during activation.

As for crack generation, different behaviors were observed in the MCXs, as mentioned previously.^27^ The MCX from R/W = 45 showed crack generation with 6 h activation, regardless of the F/R ratio (Table S3†). However, some MCXs from R/W = 40 exhibited no crack generation (F/R = 2.2 and 2.4) while others showed crack generation (F/R = 2.6 and 2.8) with 6 h activation (Table S2†). It is interesting to note that the MCX with interconnected particles showed no cracks, while those with highly aggregated particles exhibited cracks. Since those showing cracks are samples with high R/W and F/R ratios, it is believed that aggregated particles generate large residual stress upon weight loss or shrinkage, leading to the formation of cracks upon activation, in contrast to the small stress generated by interconnected particles.

When the MCXs showing crack generation after 6 h activation are excluded, only the two MCXs from F/R = 2.2 and 2.4 at R/W = 40 remain. Of these, only the latter MCX showed co-continuous pore structure with interconnected particles. Thus, this sample was selected for the N_2_ sorption study, which resulted in a Type I isotherm after activation. This is similar to the result obtained before activation, but the adsorbed volume of N_2_ increased dramatically with activation time (Fig. 2e). This is due to the increased micro- and meso-pores upon activation, which was confirmed by pore size distribution (Fig. 2f), as well as micro-pore volume measurements (Table 1). As expected,^18,22,37^ the activation resulted in high SSA values of 1481, 2489 and 3418 m^2^ g^−1^ for 2, 4 and 6 h, respectively, which are much higher than 641 m^2^ g^−1^ obtained from the MCX before activation.

**Fig. 3:**
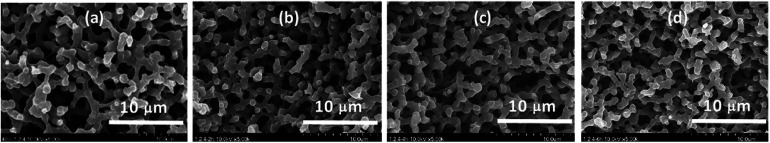
FE-SEM micrographs of RF xerogels after activation for F/R = 2.4 at R/W = 40 for 0 h (a), 2 h (b), 4 h (c), and 6 h (d).

According to the FE-SEM analysis, the pore structure of MCXs after activation is similar to each other, irrespective of the activation time (Fig. 3), and also similar to the structure before activation, as reported previously.^27,37^ This indicates that there was no shrinkage upon activation, which appears to be inconsistent with the results of large BOR and size reduction upon activation. It is believed that the size reduction arises from the removal of the outer layer of MCX by CO_2_ oxidation, which is also partially responsible for the large BOR.^27^ Of course, the large BOR is also believed to be caused by micro- and meso-pore formation in the skeleton upon activation, which in turn increases the SSA dramatically, but this cannot be demonstrated by FE-SEM.

## Conclusion

Monolithic carbon xerogel (MCX) with co-continuous hierarchical pore structure was successfully prepared *via* one-step, catalyst-free and template-free hydrothermal reaction with resorcinol (R) and formaldehyde (F), followed by pyrolysis and CO_2_ activation. A pore structure with interconnected carbon particles was obtained only from F/R = 2.4 and 2.6 at R/W = 40, as well as F/R = 2.2 at R/W = 45, while a structure with aggregated particles was obtained from others. The hydrothermal reaction is also believed to have generated a pore structure that is hard enough to provide xerogels, without having supercritical drying. Upon 6 h CO_2_ activation, all xerogels showed cracks except the one from F/R = 2.4 at R/W = 40. When this xerogel was subjected to a N_2_ sorption study after CO_2_ activation of 2, 4 and 6 h, Type I isotherms with SSA of 1481, 2489 and 3418 m^2^ g^−1^ were obtained, respectively. A comparison of these results with the Type I isotherm and SSA of 641 m^2^ g^−1^ obtained before activation demonstrates a great increase in the SSA, which is attributed to the introduction of micro- and meso-pores *via* activation, as confirmed by the pore size distribution. The FE-SEM analysis revealed a similar pore structure before and after 6 h activation, despite the large weight loss upon activation resulting from the high-degree oxidation of the outer layer, as evidenced by the large volume shrinkage upon activation.

## Conflicts of interest

There is no conflict to declare.

## Supplementary Material

RA-009-C9RA00904C-s001
