# Supplementary material for: Monolithic carbon xerogel with co-continuous hierarchical porosity via one-step, template- and catalyst-free hydrothermal reaction with resorcinol and formaldehyde
Source: RSC Adv. 2019 Mar 26;9(17):9480–5. doi: 10.1039/c9ra00904c (PMC9062128; doi:10.1039/c9ra00904c)
Supplement: RA-009-C9RA00904C-s001 [file RA-009-C9RA00904C-s001.pdf]

Electronic Supplementary Material (ESI) for RSC Advances.
This journal is © The Royal Society of Chemistry 2019

Electronic Supplementary Information (ESI)

Monolithic Carbon Xerogels via with Co-continuous Hierarchical Porosity via One-step, Template- and Catalyst-Free Hydrothermal Reaction with Resorcinol and Formaldehyde

Hyoung-Ju Yoon, Jae Young Lee and Jae-Suk Lee Tae-Ho Yoon*

School of Materials Science and Engineering, Gwangju Institute of Science and Engineering, Gwangju, 61005, South Korea.

* To whom correspondence should be addressed (thyoon@gist.ac.kr, Fax: +82-62-715-2304, Tel:+82-62-715-2307)

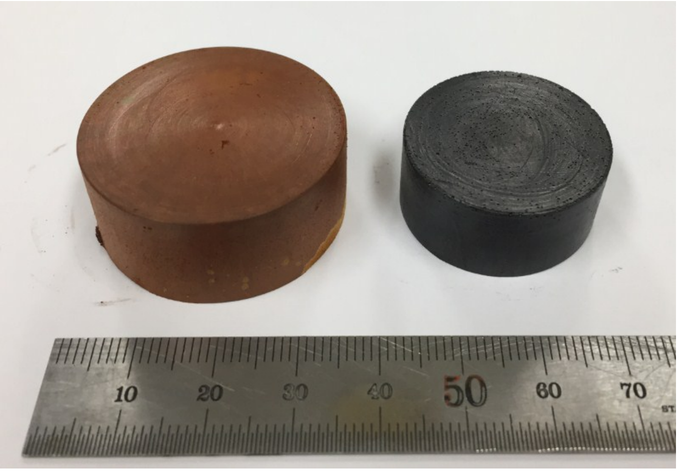

Fig. S1. Photo of monolithic RF (left) and carbon (right) xerogel.

Table S1. Characteristics of monolithic carbon xerogels at R/W=45.

Sample	S^a (m^2 g^{-1})	V_{total}^b (cm^3 g^{-1})	V_{micro}^c(cm^3 g^{-1})	D_p^d (nm)
45-2.2	633	0.247	0.225(91.1%)	1.61
45-2.4	631	0.292	0.221(75.7%)	1.85
45-2.6	623	0.352	0.216(61.4%)	2.26
45-2.8	611	0.398	0.209(52.5%)	2.76

[a]Specific surface area by BET (Brunauer-Emmett-Teller) method.

[b]Total pore volume obtained by total single point adsorption of the pores less than 300nm at *P/Po*=0.99.

[b]Micro-pore volume obtained by *t*-plot method.

[d]Pore size distribution calculated by BJH(Barrett-Joyner-Halenda) method.

Table S2. Burn off ratio (%) of monolithic carbon xerogels from F/R of 2.2, 2.4, 2.6 and 2.8 at R/W=40 upon activation.

	2.2	2.4	2.6	2.8
2 h	37	35	33	29
4 h	63	60	57	(48)
6 h	80	79	(77)	(70)

( ) : crack generation upon activation

Table S3. Burn off ratio (%) of monolithic carbon xerogels from F/R of 2.2, 2.4, 2.6 and 2.8 at R/W=45 upon activation.

	2.2	2.4	2.6	2.8
2 h	32	30	29	27
4 h	59	55	(51)	(48)
6 h	(73)	(70)	(69)	(64)

( ) : crack generation upon activation
